# A Content Incontinent: Report of Liposomal Bupivacaine Induced Fecal Incontinence

**DOI:** 10.1155/2016/7164983

**Published:** 2016-09-22

**Authors:** Emanuel A. Shapera, Vinay K. Rai

**Affiliations:** ^1^Department of Surgery, Mountain View Hospital, Las Vegas, NV, USA; ^2^Department of Surgery, University of New Mexico, Albuquerque, NM, USA

## Abstract

Proper surgical management of anal fistula demands sound clinical judgment and extraordinary care to prevent incontinence and adequate postoperative pain control and provide satisfactory resolution to optimize quality of life. Fecal incontinence can be a devastating complication of procedures performed for* fistula in ano*. We report a unique case in which temporary incontinence (for less than 4 days) followed injection of liposomal bupivacaine for postoperative pain control after draining seton placement for* fistula in ano*. Patients and physicians should be aware as it may be mistaken for a more serious anatomical and permanent cause of fecal incontinence.

## 1. Introduction

Surgical procedures in the perianal region can generate significant postoperative pain. Liposomal bupivacaine offers long-acting local analgesia that may improve patient satisfaction when administered intraoperatively [1]. However, this agent may also result in extensive sacral motor and sensory nerve block to the extent that fecal incontinence ensues; this case report aims to empower patients by highlighting this possibility so that physicians and patients can be informed should this complication occur.

## 2. Case Report

**Figure 1:**
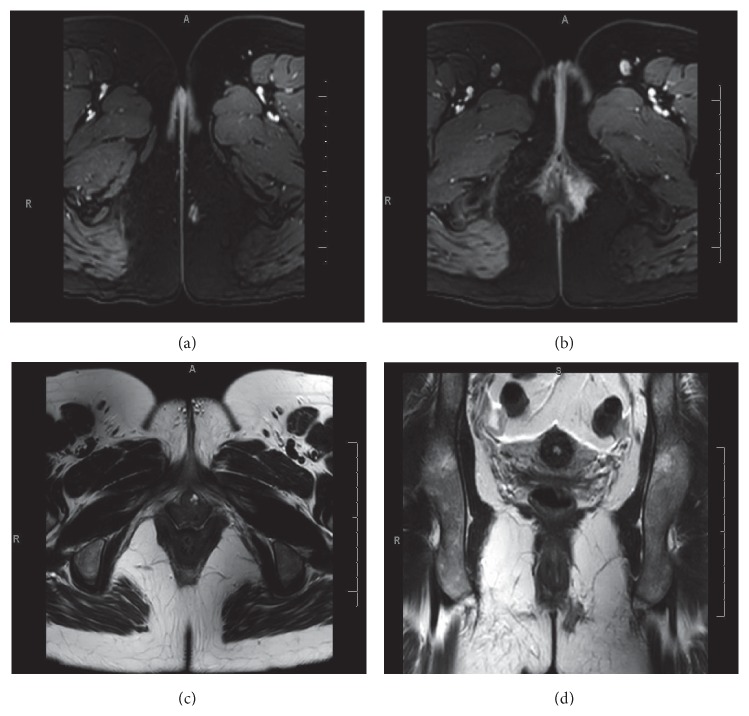
(a) T1 weighted axial slice revealing caudalmost extent of fistula, (b) T1 weighted axial slice revealing rostral extent with subcutaneous tract in an anteromedial direction, and (c) subcutaneous opening to fistula tract on T2 weighted axial slice. (d) Coronal T2 weighted slice of the fistula tract.

A 35-year-old female presented to clinic with an intermittently painful sensation of a perianal mass and occasional rectal bleeding for the past three months. On exam, a raised perianal lesion with a cord-like structure suggestive of a fistula tract was palpated. At the time of presentation, the patient did not have an abscess and an external opening was not present. Pelvic MRI revealed a left-sided anal fistula without abscess (Figure 1). She was taken to the operating room for colonoscopy, examination under anesthesia, and draining seton placement. Intraoperatively, an incision was made over the suspected lesion, which consisted of granulation, fibrous and adipose tissue at the end of the sinus tract. Elastic band setons were placed for a left anterior transsphincteric perianal fistula. Liposomal bupivacaine (20 mL) was injected in the left anterior quadrant at the incision site. Colonoscopy was unremarkable. The patient did well postoperatively, voided, and was discharged with oxycodone medication. On postoperative day 1, the patient reported satisfactory pain control but complained of complete fecal incontinence and numbness in the perianal region. On examination, the patient had saddle anesthesia with extension of sensory loss to the medial upper thighs bilaterally. On postoperative day 5, the patient reported that while her saddle sensation had returned along with fecal continence, her postoperative pain had increased. She recovered and eight weeks postoperatively underwent successful endorectal flap advancement for definitive therapy and seton removal. She reported adequate recovery without neurologic deficits two weeks postoperatively from her last surgical procedure.

## 3. Discussion

Fecal incontinence following fistulotomy, secondary to separation of the fibers of the anal sphincter, can severely compromise a patient's quality of life [2]. The temporary nature of this patient's incontinence suggested a nonanatomical etiology. Since the duration of action of liposomal bupivacaine is approximately four days [1], the patient's simultaneous resolution of incontinence, saddle anesthesia, and increase in postoperative pain within that time frame suggests it was the unusual and unique etiology of the patient's incontinence rather than a feared postoperative complication of damage to the sphincter. On the other hand, injection into a single quadrant is unlikely to result in bilateral pudendal nerve block.

Another possible contributing cause is myotoxicity to the sphincter muscles from sustained bupivacaine release, as demonstrated by Padera et al. [3]. The investigators injected bupivacaine of various lipid and nonlipid bound formulations into rats and tested motor and sensory innervation. They determined that Bupivacaine's sustained release and its greatest time-dependent concentration resulted in the greatest myotoxicity rather than any initial release burst. However, motor function was lost for a greater duration than sensory innervation, unlike in our patient where it was lost and regained at the same time. Furthermore, toxic effects last for 2 weeks to up to 1 month; in our patient they resolved in 4 days. The distribution of sensory loss to the upper medial thighs bilaterally argues against this mechanism as the sole cause.

Exparel*™*, the trademark name for liposomal bupivacaine, has been FDA approved for postsurgical pain control. Bupivacaine, an amide local anesthetic, is contained within lipid-bound spheres which slowly and spontaneously release their contents, resulting in a constant release of medication over four days. Concomitant use with lidocaine can result in early release of bupivacaine and is contraindicated within 20 minutes of administration of each other [4], which was not used in our case. Although not mentioned by the manufacturer, liposomal bupivacaine should be injected into four quadrants. We injected it in one quadrant around the external opening where we believed the source of the patient's pain would be; the fact that this resulted in complete fecal incontinence is remarkable, as it would suggest bilateral pudendal nerve block. Prospective randomized trials have demonstrated efficacy versus placebo in postoperative pain control after bunionectomy and hemorrhoidectomy [5], suggesting a potential role in postoperative pain control after perianal procedures, such as those following management of perianal fistulas. Another study comparing its effects against short-acting bupivacaine concluded there was no clinically significant benefit [6]. A 2014 study in 861 patients, including 176 undergoing hemorrhoidectomy, did not report fecal incontinence as an adverse effect [7]. Liposomal bupivacaine use is contraindicated as a paracervical block in obstetrical procedures, because it can cause serious fetal bradycardia and death [8]; it has not been reported to cause a cauda-equina syndrome in the mother, as it did cause in our patient with numbness and fecal incontinence. There is no mention in the literature suggesting liposomal bupivacaine causing fecal incontinence; we report the first such case.

## 4. Conclusion

Liposomal bupivacaine can be effective analgesia postoperatively with satisfactory pain control reported by our patient. However, its use in the perianal region may precipitate reversible fecal incontinence. Our patient recovered without any permanent neuromuscular deficits in the perineum. Physicians must counsel their patients about this potential complication.

## References

[1] Chahar P., Cummings K. C. (2012). Liposomal bupivacaine: a review of a new bupivacaine formulation. *Journal of Pain Research*.

[2] Schwartz S. I., Brunicardi F. C., Andersen D. K. (2015). Fistula in ano. *Schwartz's Principles of Surgery*.

[3] Padera R., Bellas E., Tse J. Y., Hao D., Kohane D. S. (2008). Local myotoxicity from sustained release of bupivacaine from microparticles. *Anesthesiology*.

[4] Richard B. M., Rickert D. E., Doolittle D., Mize A., Liu J., Lawson C. F. (2011). Pharmacokinetic compatibility study of lidocaine with EXPAREL in yucatan miniature pigs. *ISRN Pharmaceutics*.

[5] Gorfine S. R., Onel E., Patou G., Krivokapic Z. V. (2011). Bupivacaine extended-release liposome injection for prolonged postsurgical analgesia in patients undergoing hemorrhoidectomy: a multicenter, randomized, double-blind, placebo-controlled trial. *Diseases of the Colon and Rectum*.

[6] Nadeau M. H., Saraswat A., Vasko A., Elliott J. O., Vasko S. D. (2016). Bupivacaine versus liposomal bupivacaine for postoperative pain control after augmentation mammaplasty: a prospective, randomized, double-blind trial. *Aesthetic Surgery Journal*.

[7] Viscusi E. R., Sinatra R., Onel E., Ramamoorthy S. L. (2014). The safety of liposome bupivacaine, a novel local analgesic formulation. *Clinical Journal of Pain*.

[8] Kuczkowski K. M. (2004). Severe persistent fetal bradycardia following subarachnoid administration of fentanyl and bupivacaine for induction of a combined spinal-epidural analgesia for labor pain. *Journal of Clinical Anesthesia*.

